# Thiel’s embalming method with additional intra-cerebral ventricular formalin injection (TEIF) for cadaver training of head and brain surgery

**DOI:** 10.1007/s12565-020-00545-z

**Published:** 2020-04-27

**Authors:** Shigeta Miyake, Jun Suenaga, Ryohei Miyazaki, Jo Sasame, Taisuke Akimoto, Takahiro Tanaka, Makoto Ohtake, Hajime Takase, Kensuke Tateishi, Nobuyuki Shimizu, Hidetoshi Murata, Kengo Funakoshi, Tetsuya Yamamoto

**Affiliations:** 1 Department of Neurosurgery, Yokohama City University, 3-9 Fukuura, Kanazawa, Yokohama 2360004 Japan; 2 Department of Neuroanatomy, Yokohama City University, 3-9 Fukuura, Kanazawa, Yokohama 2360004 Japan

**Keywords:** Brain, Cadaver, Elasticity imaging techniques, Embalming, Neurosurgery

## Abstract

Thiel’s embalming method provides natural coloration, flexibility, and tissue plasticity, and is used widely to prepare specimens for cadaver surgical training. However, this method causes brain softening, thereby restricting the cadaver surgical training of intra-cranial procedures. In this study, three cadavers were embalmed using formalin fixation, Thiel’s embalming method, and Thiel’s embalming method with additional intra-cerebral ventricular formalin injection, respectively. We also established rat models of the three embalming methods to develop and determine the best method for retaining adequate brain elasticity. The intra-ventricular formalin injection in the cadaver was performed through the Kocher’s point, as in the classical external ventricular drain procedure. Both, the cadaver brains and rat models yielded consistent shear wave measurements and brain surface stiffness data. Notably, the Thiel’s embalming method with additional intra-cerebral ventricular formalin injection yielded suitable elasticity for brain cadaver surgical training in terms of brain mobilization and surgical field deployment, and also discharged formaldehyde in undetectable quantities. To our knowledge, this is the first report in which a fixed quality, namely, brain elasticity for the performance of head and brain cadaver surgical training, has been evaluated in a cadaver subjected to the Thiel’s embalming method with immersion fixation in the cerebrospinal fluid space. We conclude that the Thiel’s embalming method with additional intra-cerebral ventricular formalin injection can maintain the brain elasticity, and may therefore improve the quality of head and brain cadaver surgical training safely and easily.

## Introduction

The performance of surgical procedures requires equivalent levels of knowledge of anatomy and dissection techniques. Traditionally, anatomical studies have provided essential structural and morphological knowledge. In recent years, the introduction of various novel devices has increased the complexity of surgical procedures, and surgeons must take precautions to reduce the incidence of adverse events. Therefore, in addition to knowledge from textbooks, surgeons aim to gain practical experience with surgical maneuvers. To meet this demand, cadaver surgical training (CST) has been introduced worldwide to enable improvements in surgical techniques and outcomes (Hayashi et al. 2016).

Although CST has been performed using both, fresh and conventional formalin-fixed cadavers, certain problems have been identified (Hayashi et al. 2014). Embalming is generally required to ensure safe and reliable fixation, and to ensure a life-like tissue quality for CST, which aims to reproduce surgical techniques and views. Fresh cadavers exhibit excellent tissue preservation; however, special freezing facilities are required to prevent bacterial infection and decomposition. Traditional formalin fixation provides excellent tissue fixation and disinfection; however, it increases both the stiffness of tissues and the exposure of trainees to formaldehyde.

Previous reports have described the embalming method first introduced by Walter Thiel in 1992 as among the most suitable for CST, as it provides reliable fixation and disinfection, thereby retaining the color, flexibility, and plasticity of tissues (Thiel 2002; Liao et al. 2015; Ottone et al. 2016). The literature published up to 2016 includes 82 reports that discuss the life-like tissue, organ flexibility, and plasticity provided by this method (Ottone et al. 2016). Using shear wave echo elastography, Joy et al. (2015) found that cervical organs and muscles fixed using Thiel’s embalming method retained a life-like level of elasticity (Eisma et al. 2011, 2013; Verstraete et al. 2015).

Despite its advantages, Thiel’s embalming method causes softening of the brain (Benkhadra et al. 2011), which leads to major issues of crumbling and melting of the tissues during neurosurgical CST. Although Thiel’s embalming method enables reproducible extra-dural surgical views owing to tissue flexibility, intra-cranial surgical procedures have not been reproducible in these specimens. Here, we report the use of Thiel’s embalming method with additional intra-cerebral ventricular formalin injection (TEIF), which aimed to improve brain plasticity and tissue flexibility.

## Materials and methods

All cadavers used in this study were donated in accordance with living wills and the informed consent of bereaved family members, and this manuscript complies with Japanese acts and guidelines (the Postmortem Examination and Corpse Preservation Act, Act on Body Donation for Medical and Dental Education, and Guidelines for Cadaver Dissection in Education and Research of Clinical Medicine). Ethical approval for this study was granted by the Yokohama City University Human Ethics Committee (A1802000002) in accordance with the Guidelines for Cadaver Dissection in Education and Research of Clinical Medicine. All cadavers used in this study were prepared for a CST course intended for neurosurgeons. Anatomical photographs were obtained during CST procedures using a surgical microscope (KINEVO 900 and OPMI PENTERO 800) leased from Carl Zeiss Meditech (Jena, Germany).

### Cadaver information and embalming methods

**Fig. 1:**
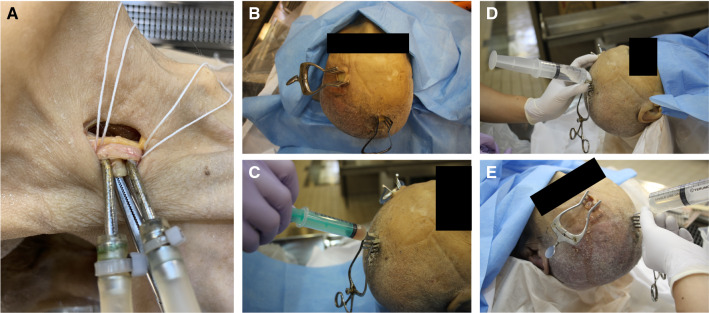
**a** The right cervical image reveals reflux fixation through the common carotid artery during Thiel’s embalming method. **b**–**e** Images of the cadaver head depict intra-ventricle formalin injection. **b** The skin incisions at the right Kocher’s point and left frontal forehead. **c** Confirmation of cerebrospinal fluid (CSF) backflow from a 16-G catheter inserted in the lateral ventricle. **d** Injection of 10% formalin into the CSF space after confirmation of CSF back flow. **e** Emission of CSF from the left forehead hole

Three cadavers were embalmed using formalin fixation, Thiel’s embalming method, and TEIF, respectively (Table 1). Formalin embalming was performed using 10% formaldehyde and a reflux fixation procedure via the femoral artery. Thiel’s embalming (Hayashi et al. 2014) was performed using solutions A, B, C, and 40% formaldehyde (the final concentration of formaldehyde was adjusted to 2%), with a reflux fixation procedure via the common carotid artery (Fig. 1a). Thiel’s embalming solution was purchased from A.S. Chemical (Osaka, Japan).

**Table 1:** Characteristics of the three cadavers

Sex	Age at death (y.o.)	Body weight (kg)	Cause of death	Embalming method	Embalming duration (days)
Female	87	43	Pneumonia	Formalin	122
Female	98	38	Senility	Thiel’s	54
Female	86	51	Pneumonia	TEIF^a^	45

^a^Theil’s embalming method with additional intra-cerebral ventricular formalin injection

TEIF was initially performed as described for Thiel’s embalming, with the addition of an intra-cerebral ventricular injection of formalin 2 days before decapitation (Fig. 1b–e). The intra-cerebral ventricular injection of formalin was performed according to the placement of the external ventricular drain via the Kocher’s point (Morone et al. 2019), which is located 11 cm superior and posterior from the nasion, and 3 cm lateral to the midline. The anterior horn of the lateral ventricle is approximately 6 cm below the Kocher’s point, and is directed at an angle drawn from the ipsilateral medial canthus and external auditory meatus. For this procedure, the cadaver was placed in the supine position, and a 1-cm skin incision and skull drilling were performed at the right Kocher’s point (Fig. 1b). A 16-G catheter was inserted into the lateral ventricle, and the back flow of cerebrospinal fluid (CSF) was confirmed (Fig. 1c). After creating a hole in the contralateral left forehead for the emission of CSF, 400 ml of 10% formaldehyde was injected into the CSF space through the catheter (Fig. 1d, e), which was then sealed with a piece of wood and glue. All cadavers were stored at 4 °C.

### Rat models of embalming

All animal procedures were performed in strict accordance with the Guide for the Care and Use of Laboratory Animals of the National Institutes of Health. The animal research protocol was approved by the Institutional Laboratory Animal Care and Ethics Committee of Yokohama City University (Approval numbers: F-A-14-109, F-A-17-044, F-A-19-025). All rats were embalmed using the reflux fixation method with cannulation into the cardiac apex, using one of the following embalming solutions (400 ml): 10% formalin, Thiel’s embalming solution (2% formaldehyde), and phosphate-buffered saline (PBS: control). A rat model of TEIF was established by subjecting the animal to Thiel’s embalming for a month, followed by the injection of 4.0 ml of 10% formaldehyde into the cerebral ventricle for two days before measurements using a previously reported stereotactic intra-cerebral ventricular injection method (Kishimoto et al. 2019). The control rat brain treated with PBS was measured immediately after treatment. Three rat models were used for each embalming method (formalin, Thiel’s, TEIF, and PBS) and embalming period (3 days and 1 month) in this study. All embalmed rats were stored at 4 °C.

### Measurement of brain elasticity and airborne formaldehyde concentration

We used three methods to evaluate the elasticity of the embalmed brains. The main assessment involved the surgical appearance of the brain in response to pressure from surgical instruments (e.g., spatula), surgical field deployment, and tissue coloration. Shear wave measurements (SWMs) were obtained via elastography scans performed using a linear-array transducer (ALOKA ARIETTA 850; Hitachi, Tokyo, Japan), which provided a B-mode image and region of interest (ROI) setting. In this analysis, we evaluated the supra and infratentorial brain structures on a single side of each human brain, avoiding sulci and coronal sections. After confirming the acceptable image and adequate ROI in the B-mode image, the SWM was obtained 10 times per area. As the SWM is easily affected by vibrations and the pressure applied by the examiner, this assessment also enables the calculation of a reliability score. In this study, reliable SWMs, defined as those with reliability scores > 20%, were used for further analysis.

The objective parameters of tissue elasticity and brain surface stiffness were measured using ultrasound sonography and a hardness meter (OB-200G, Oba Instrument Works, Tokyo, Japan), respectively. For the latter measurement, the brain was pressed using the hardness meter, and the force required to crumble the brain surface was measured up to 200 g/N. It was possible to measure the brain surface stiffness once per each site, as the brain surface was collapsed after measurement. This parameter was assessed after SWM measurement, and both measures used the same assessment points. Finally, the formaldehyde concentration in the air after the dural incision was assessed at a 30-cm distance from the head, using a formaldehyde detection tube once per each head, which could detect concentrations of 0.01–0.48 ppm (Komyo Rikagaku Kogyo, Kawasaki, Japan).

### Data analysis

All data were evaluated and analyzed using Prism 7.0 J for Windows (GraphPad Software, Inc., San Diego, CA, USA).

## Results

### Surgical views

**Fig. 2:**
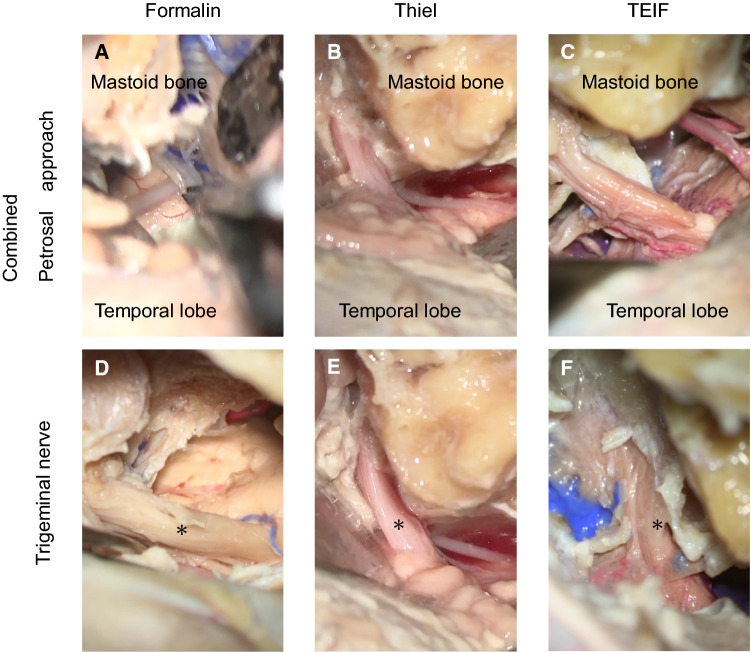
**a**–**c** Intra-cranial microscopical surgical views of the combined petrosal approach. **a** An image of a formalin-embalmed cadaver reveals a narrow and deep surgical field. **b** An image of a cadaver embalmed using Thiel’s method shows a crumbling brain. **c** An image of a cadaver fixed via Thiel’s embalming method with an additional intra-cerebral ventricular formalin injection (TEIF) reveals adequate deployment of the deep surgical field. **d**–**f** The trigeminal nerve (*) is displayed in each cadaver. **d** The formalin-fixed nerve exhibited discoloration. **e** The nerve subjected to Thiel’s embalming method had a pink color and wet texture. **f** The nerve subjected to TEIF fixation had an intermediate texture

Among all the cadavers, the TEIF-fixed cadaver provided adequate surgical fields and acceptable structural coloration, exhibiting an appropriate fixation for CST. For observing the cranial nerves, each cadaver head was subjected to a combined petrosal approach, one of the most difficult skull base approach that involves drilling the temporal bone. The formalin-fixed soft tissues and brain were too stiff, and could not be moved easily to provide adequate traction and maintain surgical views. Consequently, the formalin-fixed cadaver provided surgical fields that were too narrow and deep, requiring reduction of the peaked brain (Fig. 2a). The soft tissues in the cadaver subjected to Thiel’s embalming were flexible and easy to manipulate; however, the brain exhibited obvious softening and crumbled easily under traction. Although this method provided wide surgical views, the deep structures could not be deployed (Fig. 2b). The soft tissues in the TEIF-fixed cadaver remained flexible, and the brain retained adequate elasticity and surface durability. Therefore, this technique provided a life-like surgical field (Fig. 2c).

Structural coloration was evaluated by observing the trigeminal nerve. In the combined petrosal approach, the trigeminal nerve, a thick structure, is observed in the center of the surgical field. In the formalin-embalmed head, the nerve was discolored, with a solid texture. In the head embalmed using Thiel’s method, the nerve retained a natural color, but exhibited a moist texture. The TEIF-fixed nerve exhibited an intermediate condition between those achieved with the other techniques (Fig. 2d–f).

### Elasticity of the cadaver brain

**Fig. 3:**
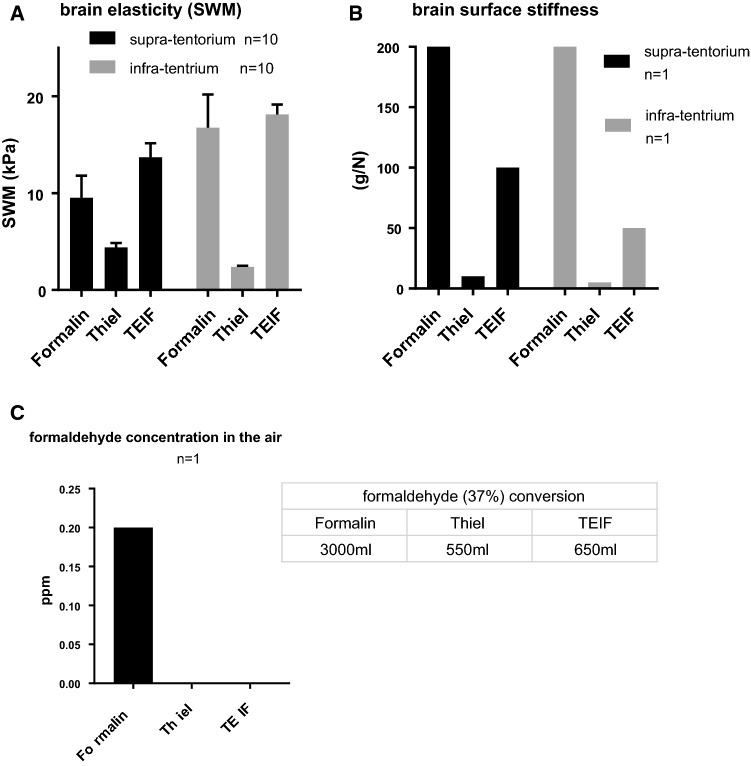
**a** Shear wave measurements obtained from the supra (black bar) and infra (gray bar) tentorium of each embalmed cadaver brain (*n* = 10). The bars show standard errors of the mean. **b** Stiffness of the brain surface in the supra (black bar) and infra (gray bar) tentorium of each embalmed cadaver (*n* = 1). **c** Airborne formaldehyde concentration detected at a 30-cm distance from each cadaver brain (*n* = 1). The amounts of formalin (37% concentration) used for each embalming method are listed below the graph

The objective analysis of brain elasticity revealed that the TEIF method enabled a better recovery of brain plasticity than the Thiel’s method. The SWM demonstrated that the brain embalmed using Thiel’s method was softer than the TEIF-fixed brain, which regained elasticity on both sides of the tentorium (Fig. 3a). The analysis of brain surface stiffness revealed a value > 200 g/N in the formalin-fixed brain, whereas both sides of the tentorium in the brain fixed using Thiel’s embalming method were vulnerable to pressure. The TEIF-fixed brain exhibited stiffness values of 100 and 50 g/N in the supra-tentorium and infra-tentorium (Fig. 3b). Moreover, TEIF fixation released a safe amount of formaldehyde into the air (Fig. 3c). The airborne formaldehyde concentration associated with the formalin fixation, Thiel’s embalming method, and TEIF fixation were 0.20, < 0.01, and < 0.01 ppm, respectively.

### Elasticity of rat brain

**Fig. 4:**
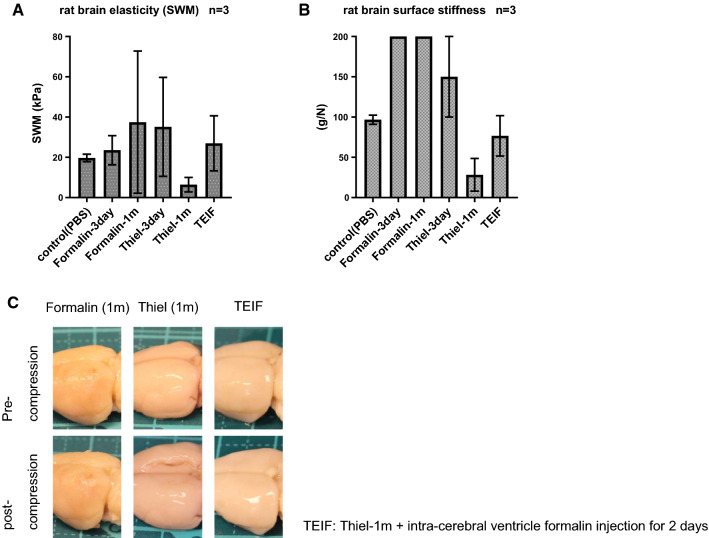
**a** Shear wave measurements (SWM) obtained from rat models of embalming (*n* = 3). The error bars show standard deviations. **b** Stiffnesses of the brain surfaces in rat models of embalming (*n* = 3). The error bars show standard deviations. **c** Pre- and post-compression status of rat brains embalmed using formalin, Thiel’s method, and TEIF. The brain subjected to Thiel’s method for 1 month crumbled after compression; in contrast, the TEIF-fixed brain regained the original shape

Using the rat models, we could reproduce the SWM and surface stiffness observed in the brains of embalmed human cadavers. The model analysis also enabled us to evaluate the differences in elasticity between the embalmed and control brains, and the effect of the fixation period. In the model of formalin fixation, the SWM increased gradually relative to the control, and the brain surface stiffness was higher than the upper limit of measurement (200 g/N), beginning at 3 days after fixation. In the model subjected to Thiel’s embalming, both the SWM and brain surface stiffness increased slightly relative to the control at 3 days after fixation, but exhibited softening after 1 month. In contrast, the model of TEIF fixation exhibited recovery of the SWM and brain surface stiffness to levels similar to those in the control brain (Fig. 4a, b). Moreover, the TEIF-fixed brain recovered to the original shape after compression, whereas the brain subjected to Thiel’s embalming crumbled; the formalin-fixed brain did not deform (Fig. 4c).

## Discussion

In this study, TEIF fixation overcame the brain softening associated with Thiel’s embalming method, and therefore provided suitable conditions for head and brain CST. Moreover, the rat embalming models used in this study demonstrated significant softening of the brain after 1 month with the Thiel’s embalming method. For brain CST, which involves both skull drilling and intra-cranial procedures, adequate brain elasticity is essential for the deployment of a deep surgical field and support of the mobilized brain. Although the softening of a brain subjected to Thiel’s embalming is a major issue affecting the performance of head CST, this process has not been clearly described. Benkhadra et al. (2011) described the poor condition of the brain subjected to the Thiel’s embalming method.

We recognize that the use of the CSF space for the immersion fixation of a brain subjected to Thiel’s embalming method is actually a traditional idea. Thiel (2002) suggested the injection of a fixed solution into the CSF space through the ethmoid bone as a method of additional brain fixation. However, this proposal was not widely adopted owing to the complexity of the procedures, and the associated damage to the skull base and structures. In this study, we approached the CSF space using a previously reported simple and common external ventricle drain technique (Morone et al. 2019), and demonstrated that this procedure could successfully restore brain elasticity. Moreover, this TEIF procedure exhibited a low risk of formaldehyde exposure.

As noted, we assessed brain elasticity using two independent measures. Previously, ultrasound-based SWM elastography was reported as an effective method for measuring cadaver organ elasticity (Joy et al. 2015). In this study, we measured the SWM in a ROI located in the white matter of the brain, as a previous magnetic resonance imaging analysis of a brain subjected to Thiel’s embalming exhibited distinct areas of white and gray matter (Eljamel et al. 2014). Therefore, the SWM indicates the elasticity of white matter, which may affect the ability to mobilize the brain. We evaluated the brain surface stiffness as a measure of cortical elasticity that may affect the vulnerability of the brain tissue to crumbling. Hence, the two objective assessments of brain elasticity applied in this study may fully reflect the ability to deploy the surgical field. Accordingly, TEIF fixation yielded approximately the same level of elasticity as observed in the control brain, suggesting that this method imitates a surgical situation.

The results of our elasticity assessment indicate that TEIF is the most suitable method for brain CST in terms of deployment of the surgical field and reproducibility of the intra-cranial procedure. TEIF therefore appears to be a promising embalming method for head and brain CST. Despite these advantages, our study had certain limitations. For instance, although there were obvious differences and tendencies among the three fixations, each fixation method was tested in only one cadaver and three rats, and the ideal duration of immersion fixation remains unclear. Additional experience and further investigations on TEIF will yield more concrete evidence.

In conclusion, we found that TEIF could easily and safely overcome the brain softening associated with Thiel’s embalming. It may potentially be used to improve the quality of head and brain CST, and enhance the development of neurosurgical skills.
